# Heavy drinking and contextual risk factors among adults in South Africa: findings from the International Alcohol Control study

**DOI:** 10.1186/s13011-018-0182-1

**Published:** 2018-12-05

**Authors:** Pamela J. Trangenstein, Neo K. Morojele, Carl Lombard, David H. Jernigan, Charles D. H. Parry

**Affiliations:** 1 Alcohol Research Group, 6001 Shellmound St., Suite 450, Emeryville, CA 94608 USA; 2 Department of Health Law, Policy and Management, Boston University School of Public Health, 715 Albany St, Boston, MA 02118 USA; 3 Alcohol, Tobacco & Other Drug Research Unit, South African Medical Research Council, 1 Soutpansberg Rd, Prinshof 349-Jr, Pretoria, 0084 South Africa; 4 School of Public Health, University of the Witwatersrand, 1 Jan Smuts Avenue, Braamfontein, Johannesburg, 2000 South Africa; 5 School of Public Health and Family Medicine, University of Cape Town, Falmouth Rd, Observatory, Cape Town, 7925 South Africa; 6 Biostatistics Unit, South African Medical Research Council, Francie Van Zijl Dr, Parow Valley, Cape Town, 7501 South Africa; 7 Alcohol, Tobacco & Other Drug Research Unit, South African Medical Research Council, Box 19070, Tygerberg, PO 7505 South Africa; 8 Department of Psychiatry, Francie van Zijl Drive, Tygerberg, Cape Town, 7505 South Africa

**Keywords:** Heavy drinking, Alcohol policy, Container size, South Africa

## Abstract

**Background:**

There is limited information about the potential individual-level and contextual drivers of heavy drinking in South Africa. This study aimed to identify risk factors for heavy drinking in Tshwane, South Africa.

**Methods:**

A household survey using a multi-stage stratified cluster random sampling design. Complete consumption and income data were available on 713 adults. Heavy drinking was defined as consuming ≥120 ml (96 g) of absolute alcohol (AA) for men and ≥ 90 ml (72 g) AA for women at any location at least monthly.

**Results:**

53% of the sample were heavy drinkers. Bivariate analyses revealed that heavy drinking differed by marital status, primary drinking location, and container size. Using simple logistic regression, only cider consumption was found to lower the odds of heavy drinking. Persons who primarily drank in someone else’s home, nightclubs, and sports clubs had increased odds of heavy drinking. Using multiple logistic regression and adjusting for marital status and primary container size, single persons were found to have substantially higher odds of heavy drinking. Persons who drank their primary beverage from above average-sized containers at their primary location had 7.9 times the odds of heavy drinking as compared to persons who drank from average-sized containers. Some significant associations between heavy drinking and age, race, and income were found for certain beverages.

**Conclusion:**

Rates of heavy drinking were higher than expected giving impetus to various alcohol policy reforms under consideration in South Africa. Better labeling of the alcohol content of different containers is needed together with limiting production, marketing and serving of alcohol in large containers.

## Background

In 2011, South African adults (aged 15 years and older) consumed 9.5 l of absolute alcohol each year -- higher than the average for Africa (6.0 l) and the world (6.2 l) [1]. In 2015, alcohol was the fifth leading cause of death and disability in South Africa [2], which is likely attributable to alcohol’s role in causing sexually transmitted infections and interpersonal violence, the two leading causes of death in South Africa [3–6]. In addition, community-based samples repeatedly show atypically high prevalence of fetal alcohol spectrum disorders, ranging up to 29% [7–9]. Altogether, alcohol caused 7.1% of all deaths and 7.0% of all disability-adjusted life years in South Africa in 2000 [10], and harmful alcohol use is estimated to cost R249–280 billion each year, 10–12% of South Africa’s gross domestic product [11].

Drinking patterns shape the association between alcohol consumption and related harms, because they determine the dose of toxic effects (which cause chronic disease) and the level of intoxication (which determines risk for injuries and social problems) [12]. Many alcohol-related conditions show a dose-response relationship between volume of alcohol consumption and risk of adverse outcomes [13], suggesting that heavy drinking occasions have higher risk for both toxic effects and greater intoxication [14–16].

Heavy drinking is a pattern of consumption involving consuming large volumes of alcohol during one occasion or in a short period of time. There is no universal definition for heavy drinking, but studies generally define it using one of two thresholds: 1) 60 g of absolute alcohol for men and 40 g for women or 2) 100 g of absolute alcohol for men and 60 g for women [13]. While there are criticisms of implementing a one-size-fits-all cutoff to define heavy drinking across diverse people and cultures [17], the convergent validity of heavy drinking measures is established by their use as a proxy for identifying persons who have alcohol use disorders (AUDs) [13]. Heavy drinking works as a proxy for AUDs, because the association between the two drinking patterns is almost linear and questions about number of drinks consumed may avoid the social desirability bias that problematizes other types of questions to assess alcohol-related problems [18].

Monitoring heavy drinking trends over time may help researchers predict treatment and prevention needs at the population level. In addition, drinking patterns modify the association between per capita consumption and related harms such that countries with riskier drinking patterns tend to experience increased levels of harms from increases in consumption [19]. This implies that researchers may need to monitor drinking patterns in order to accurately anticipate the potential outcomes of policies that could alter per capita consumption.

South Africa is currently considering a liquor amendment bill to reduce per capita consumption, including provisions that would raise the national minimum legal purchase age from 18 to 21 years, establish a minimum 500-m buffer between alcohol outlets and other outlets or sensitive locations (e.g., schools, places of worship), and hold alcohol manufacturers and suppliers of alcohol to unlicensed alcohol outlets liable for damages resulting from consumption of their products. The proposed bill also brings some informally and illicitly produced alcohol into the regulated sector by lowering the threshold used to define alcoholic beverages from 1.0% alcohol to 0.5% alcohol [20].

However, there is an incomplete picture of heavy drinkers in South Africa. To date, the literature begins to assemble demographic profiles of heavy drinkers: they tend to be young, male, Black African or Coloured, and reside in urban areas [21, 22]. However, researchers need more than a simplistic analysis of demographics to understand the possible push factors that promote heavy drinking and serve as potential points of intervention. To the best of our knowledge, there is no research about the contextual factors surrounding heavy drinking in South Africa. Given this, the present analysis aims to describe the demographic characteristics of heavy drinkers, where they primarily drink, the type of alcohol they consume most often, and the container size that they typically drink from in the Tshwane Metropole. Secondary analyses aim to determine the distribution of consumption by decile of drinker and identify characteristics of heavy drinking occasions.

## Materials and methods

### Sample and data collection

Data for this study are from the South African arm of the multi-country International Alcohol Control (IAC) study [23]. This cross-sectional study was conducted during 2014 in the Tshwane Metropole, located around the executive capital, Pretoria. It is located mainly within the province of Gauteng and overlaps into part of North West province. It consists of five regions and 76 wards. The estimated population of Tshwane is 3.3 million [24].

The study used a multi-stage stratified cluster random sampling design. There were four stages to the cluster random sampling involving sampling of wards (Stage 1), enumeration areas (EAs) within selected wards (Stage 2), households within selected EAs (Stage 3), and study participants within selected households (Stage 4). This is described in detail elsewhere [25]. Data were weighted to take into account the underlying structure of the realized sample and the sample frame to ensure a random selection of respondents. Eligible participants had to have consumed alcohol in the past six months and be 18 to 65 years old. The target sample size of 2000 was determined by the IAC Study [23]. The overall response rate was 78% [25].

### Measures used in this analysis

We adapted the standard (English) IAC questionnaire, then translated and back-translated it into seTswana and Afrikaans. It included various items, with those relevant to this paper being demographic factors (e.g., age, gender, total annual personal income, and marital status) and alcohol consumption.

#### Sociodemographic variables

Participants’ ages were categorized as: 18–19, 20–24, 25–34, 35–44, 45–54, and 55–65 (reference group). Annual personal income was categorized into low (<R30,000 - reference group), medium (>R30,000 but ≤R200,000), and high (>R200,000).

#### Heavy drinking

Heavy drinking was defined as consuming 96 g of absolute alcohol (AA) or more (roughly 8 standard drinks, or 120 ml) for men or 72 g or more (roughly 6 standard drinks, or 90 ml) for women at any location at least monthly. This definition, used by the IAC study [26], is higher than typically used in surveys and by the WHO, but reflects a growing questioning of the validity of the 4+/5+ binge or heavy drinking criterion [17]. The questionnaire asked quantity and frequency of typical alcohol consumption at each of 16 locations (i.e., your home, someone else’s home, nightclubs, other clubs, restaurants, theatres, workplaces, planes, motor vehicles, sports events, outdoors, shebeens, bars, hotels, special events, and other) over the past six months. We then calculated absolute alcohol for each beverage type as (number of containers)*(container size)*(percent absolute alcohol) by location. The absolute alcohol for each beverage type and location was then summed to determine average consumption of AA by location. The heavy drinking variable was dichotomous and separated persons who reported consuming more than 96 g (for men) or 72 g (for women) of AA on an average occasion at least monthly from those who did not (reference group).

#### Heavy drinking occasions

Each typical drinking occasion at each location was classified as low risk or heavy drinking based on the usual quantity of alcohol consumed at least monthly.

Occasions that did not include heavy drinking (96 g AA for men and 72 g AA for women) were defined as low risk.

#### Primary drinking location

Primary drinking location was defined as the location in which the participant reported drinking most frequently. If the participant drank at two locations with the same maximum frequency, then the location where the participant consumed a greater quantity of absolute alcohol was selected. If there were two locations with the same maximum frequency and quantity, then the more exotic location was selected (e.g., nightclubs and special events are more exotic than homes and restaurants). The primary drinking location variable was categorical with 12 of the 16 original drinking locations included: own home, someone else’s home, nightclubs, sports clubs, other clubs, restaurants, motor, sports events, outdoors, shebeen, pub, hotels, special events, and other. No participants primarily drank in theaters, planes, workplaces, hotels, or at sports events.

#### Primary beverage

The primary beverage consumed at the primary drinking location was selected by determining the beverage the participant drank with maximum quantity (of AA) at that location. The primary beverage variable was categorical with 12 of the original 14 beverage types: beer; low alcohol beer; home brew beer; stout; wine; spirits; cocktails; liqueur; shooters; sherry, port, or vermouth; cider; alcopops (a ready-mixed drink that resembles a soft drink but contains alcohol), and other beverages. No participants primarily drank other beverages or sherry, port, or vermouth.

#### Container size

Container size was determined as the usual container size of the primary beverage at the primary drinking location, and was categorized into average, below average, or above average. Average container size was defined as the container size closest to a standard drink (i.e., 330 ml for beer; 330 ml for low alcohol beer; 500 ml for home brew beer; 330 ml for stout; 150 ml for wine; 30 ml for spirits; 30 ml for cocktails; 50 ml for liqueur; 25 ml for shooters; 50 ml for sherry, port, or vermouth; 330 ml for cider; 330 ml for alcopops; and 330 ml for other alcohols).

### Procedures

After obtaining informed consent, participants were interviewed in their homes by trained interviewers. Interviews were administered on a tablet. This approach was adopted due to the complexity of the questionnaire. After the interview, participants received a resource card for alcohol-related problems as well as a shopping or a cellular telephone recharge voucher worth R30. The study was approved by the Research Ethics Committee of the South African Medical Research Council.

### Statistical analyses

Taylor series linearization approximations [27] were used to account for the complex multi-stage sampling as implemented in the “svy” prefix in Stata version 14.0 [28]. As part of exploratory analyses, deciles of drinkers were obtained using the total amount of absolute alcohol the participant consumed across all locations and beverage types over a six-month period. The total amount of absolute alcohol consumed by each decile was summed and divided by the total amount of alcohol consumed by the entire sample to determine the percent of consumption by decile. Corrected weight chi-square tests were used to detect significant relationships between heavy drinking and the sociodemographic and alcohol consumption characteristics.

The analysis then used multivariate logistic regression to test the hypotheses that heavy drinking differs by demographics and alcohol consumption characteristics. Variables with significant relationships to the outcome variables and key demographic variables (i.e., age, gender, race/ethnicity, and total annual personal income) were selected using best subset variable selection methods with no variables forced into the model. The model included age, race/ethnicity, marital status, and container size. While other variables explained variability better than primary beverage in the main model, simple logistic regressions of heavy drinking on primary beverage type and primary drinking location were performed to investigate the impact of beverage and location choices. The multiple logistic regression model was then repeated for the persons who consumed the four main types of alcohol (i.e., beer, wine, spirits, and cider) to determine whether the associations differed by beverage. Multicollinearity was assessed by examining correlations between predictors. No two predictors had a correlation > 0.5. Model fit was checked using an adaptation of Hosmer Lemeshow’s Goodness of Fit Test, and all models indicated appropriate fit. *P* values less than 0.05 were considered statistically significant.

Nine hundred and eighty-seven participants did not report frequency data for all drinking locations, and seven participants did not report enough consumption information for their primary drinking location to determine primary beverage and/or primary container size. In addition, 449 participants did not provide a total annual personal income. These participants were excluded from the analyses. The final sample size included 713 adults.

Participants with missing consumption data did not differ from the sample on race/ethnicity (*F*_*2.06, 39.22*_ = 2.18, *p* = 0.12), income (*F*_*1.81, 34.48*_ = 0.02, *p* = 0.97), or urbanicity (*F*_*1, 19*_ = 1.17, *p* = 0.29). Participants with missing consumption data were more likely to be younger (*F*_*3.85, 73.06*_ = 4.67, *p* < 0.01) and female (*F*_*1, 19*_ = 4.71, *p* = 0.04), and missingness differed by marital status (*F*_*4.04, 76.74*_ = 5.17, *p* < 0.001). Participants with missing personal income data did not differ on gender (*F*_*1, 19*_ = 0.01, *p* = 0.92), urbanicity (*F*_*1, 19*_ = 0.32, *p* = 0.58), heavy drinking status (*F*_*1, 19*_ = 0.58, *p* = 0.46), primary beverage (*F*_*5.97, 113.36*_ = 1.96, *p* = 0.08), primary drinking location (*F*_*6.44, 122.38*_ = 1.57, *p* = 0.16), or primary beverage container size (*F*_*1.44, 27.32*_ = 2.92, *p* = 0.09). Participants with missing personal income data were more likely to be younger (*F*_*2.26, 42.99*_ = 7.24, *p* = 0.001) and less likely to be Black African (*F*_*2.10, 39.97*_ = 10.07, *p* < 0.001), and missingness differed by marital status (*F*_*3.61, 68.62*_ = 49.11, *p* < 0.001).

## Results

### Demographics & drinking characteristics

The mean age in the sample was 36.3 years, 65.8% were male, 79.1% were Black African, and 77.0% were low-income (see Table 1). Fifty-three percent of the sample were heavy drinkers. Heavy drinking did not vary by gender (*F*_*1, 19*_ = 3.96, *p* = 0.06), age (*F*_*3.86, 73.33*_ = 1.07, *p* = 0.37), race/ethnicity (*F*_*1.70, 32.34*_ = 2.51, *p* = 0.10) or total annual personal income (*F*_*1.82, 34.4*_ = 0.11, *p* = 0.87). Heavy drinking differed by marital status (*F*_*2.48, 47.11*_ = 3.09, *p* = 0.04).

**Table 1:** Characteristics of participants and comparison by drinking level

	Not Heavy Drinker (*n* = 319) % (95% CI)	Heavy Drinker (*n* = 394) % (95% CI)	*P*-Value	Total (*n* = 713) % (95% CI)
Gender			0.06	
Male	60.9 (53.4, 67.9)	70.3 (63.8, 76.1)		65.8 (61.0, 70.4)
Female	39.1 (32.1, 46.6)	29.7 (23.9, 36.2)		34.2 (29.6, 39.0)
Age			0.37	
18–19	3.1 (1.0, 8.9)	1.5 (0.5, 4.2)		2.3 (0.9, 5.3)
20–24	18.1 (12.2, 26.0)	18.1 (13.5, 23.9)		18.1 (14.3, 22.7)
25–34	26.1 (20.1, 33.2)	34.4 (27.3, 42.2)		30.5 (26.2, 35.1)
35–44	23.6 (18.3, 30.0)	22.4 (17.6, 28.1)		23.0 (19.2, 27.3)
45–54	19.2 (11.6, 30.1)	12.4 (8.3, 18.1)		15.6 (11.3, 21.1)
55–65	9.9 (5.9, 16.0)	11.3 (6.4, 19.1)		10.6 (7.3, 15.2)
Race/Ethnicity			0.10	
Black African	74.3 (62.4, 83.5)	83.4 (74.7, 89.5)		79.1 (72.3, 84.5)
Coloured	3.6 (2.2, 5.8)	6.0 (3.5, 10.0)		4.8 (3.2, 7.3)
White	21.6 (12.6, 34.4)	9.4 (4.0, 20.8)		15.2 (9.7, 23.0)
Asian/Indian	0.5 (< 0.01, 03.1)	1.3 (0.4, 4.4)		0.9 (0.3, 2.4)
Marital Status			0.04	
Married	49.7 (39.8, 59.7)	30.8 (24.3, 38.1)		39.7 (33.6, 46.2)
Co-habitating	8.1 (4.2, 14.9)	6.4 (4.4, 9.3)		7.2 (5.0, 10.3)
Never married	38.1 (30.5, 46.4)	55.6 (46.6, 64.3)		47.3 (41.3, 53.5)
Divorced	2.8 (1.2, 6.1)	3.7 (0.9, 13.9)		3.3 (1.5, 6.8)
Separated	0.8 (0.3, 1.9)	1.7 (0.8, 3.5)		1.3 (0.7, 2.4)
Widowed	0.6 (0.2, 2.2)	1.8 (0.6, 5.3)		1.2 (0.5, 3.1)
Total Annual Personal Income			0.87	
Low	77.6 (68.5, 84.7)	76.4 (69.2, 82.4)		77.0 (71.9, 81.4)
Medium	14.7 (9.3, 22.5)	16.6 (11.8, 23.0)		15.7 (12.8, 19.2)
High	7.7 (4.4, 13.1)	7.0 (4.1, 11.6)		7.3 (4.9, 10.7)
Container Size			< 0.001	
Below Average	13.1 (7.2, 22.8)	2.8 (1.1, 6.6)		7.7 (4.3, 13.3)
Average	52.6 (44.5, 60.6)	17.2 (12.4, 23.5)		34.0 (28.9, 39.6)
Above Average	34.3 (24.9, 45.1)	80.0 (72.5, 85.8)		58.3 (50.6, 65.7)
Primary Location			0.02	
Home	67.8 (58.3, 76.1)	52.8 (44.3, 61.2)		59.9 (52.7, 66.8)
Someone Else’s Home	10.7 (6.3, 17.5)	18.5 (12.7, 26.1)		14.8 (10.4, 20.6)
Nightclub	0.9 (0.3, 2.8)	3.3 (1.7, 6.3)		2.2 (1.2, 3.9)
Sports Club	< 0.01 (< 0.01, 0.5)	1.1 (0.3, 4.2)		0.6 (0.2, 2.3)
Other Club	0.6 (0.2, 2.4)	1.9 (0.9,4.2)		1.3 (0.7, 2.3)
Restaurant	1.8 (0.6, 5.0)	0.4 (< 0.01, 2.2)		1.1 (0.4, 2.5)
Motor	< 0.01 (< 0.01, 0.6)	0.6 (< 0.01, 4.6)		0.3 (< 0.1, 2.2)
Outdoors	2.5 (1.0, 6.2)	1.4 (0.4, 5.3)		1.9 (0.9, 4.0)
Shebeen	1.4 (0.5, 3.8)	2.0 (0.8, 4.8)		1.7 (0.9, 3.2)
Pub	13.8 (8.7, 21.2)	16.7 (11.7, 23.3)		15.3 (12.0, 19.5)
Special Events	0.1 (< 0.01, 1.2)	1.4 (0.2, 9.1)		0.8 (0.1, 4.4)
Other	0.2 (< 0.01, 1.2)	0		< 0.01 (0.0, 0.6)
Primary Beverage			0.10	
Beer	38.4 (30.9, 46.5)	57.5 (47.4, 67.0)		48.4 (42.1, 54.9)
Low Alcohol Beer	5.8 (2.4, 13.4)	1.1 (0.2, 7.1)		3.3 (1.5, 7.1)
Home Brew Beer	1.2 (0.4, 3.9)	0.8 (0.1, 4.3)		1.0 (0.4, 2.3)
Stout	0.5 (0.1, 2.2)	0.9 (0.4, 2.3)		0.7 (0.3, 1.6)
Wine	15.0 (8.5, 25.0)	13.9 (9.4, 20.1)		14.4 (10.8, 19.0)
Spirits	15.3 (8.0, 27.2)	10.2 (4.7, 21.0)		12.6 (6.9, 22.1)
Cocktails	< 0.01 (< 0.01, 0.5)	─^a^		< 0.1 (< 0.1, 0.2)
Liqueur	0.6 (< 0.01, 4.2)	─^a^		0.3 (< 0.1, 2.0)
Shooters	0.6 (< 0.01, 4.4)	─^a^		0.3 (< 0.1, 2.1)
Cider	20.5 (12.8, 31.1)	15.6 (11.0, 21.6)		17.9 (12.9, 24.3)
Alcopops	2.3 (0.4, 10.9)	─^a^		1.1 (0.2, 5.4)

*CI* confidence interval

^a^ All of the participants who primarily drink cocktails, liqueur, shooters, and alcopops were not heavy drinkers

Homes (59.9%), pubs (15.3%), someone else’s home (14.8%), nightclubs (2.2%), outdoors (1.9%), shebeens (1.7%), other clubs (1.3%), and restaurants (1.1%) were the most common primary drinking locations. Heavy drinking differed by primary drinking location (*F*_*6.42, 122.04*_ = 2.48, *p* = 0.02). Among the commonly reported primary drinking locations, persons who primarily drank at special events (91.8%), in motor vehicles (87.2), other clubs (77.5), nightclubs (79.8%), shebeens (61.5%), someone else’s home (65.8%), and pubs (57.3%) had the highest percentages of heavy drinking. Persons who primarily drank at restaurants (19.5%) had the lowest percentages of heavy drinking.

Beer (48.4%), cider (17.9%), wine (14.4%), and spirits (12.6%) were the most commonly reported primary beverages consumed at the primary drinking location. Heavy drinking did not differ by primary beverage (*F*_*4.92, 93.50*_ = 1.89, *p* = 0.10). High percentages of persons who primarily drank beer (62.5%), wine (50.7%), cider (45.8%), and spirits (42.7%) were heavy drinkers.

The container size of the primary beverage at the primary drinking location was also associated with heavy drinking (*F*_*1.72, 32.76*_ = 34.72, *p* < 0.001). Fifty-eight percent of the sample primarily drank from above-average sized containers, 34.0% drank from average-sized containers, and 7.7% drank from below average-sized containers. Seventy-two percent of persons who drank from above average-sized containers were heavy drinkers, while only 26.7% of persons who drank from average-sized, and 19.0% of persons who drank from below average-sized containers were heavy drinkers.

### Drinks by decile

**Fig. 1:**
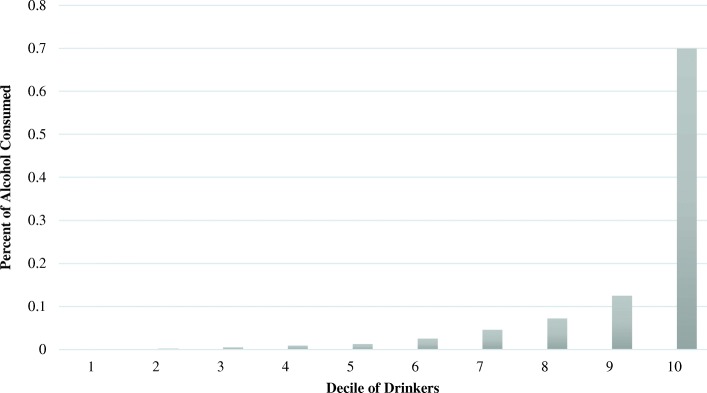
Percent of absolute alcohol consumed by decile of drinkers

The top 10% of drinkers drank 70.3% of the absolute alcohol, and the top 20% of drinkers drank 82.3% of the absolute alcohol (see Fig. 1). Together, heavy drinkers drank 93.9% of the absolute alcohol.

### Heavy drinking

Table 2 summarizes the results of the simple logistic regression predicting heavy drinking by the primary beverage. Primarily drinking cider was found to lower the odds of heavy drinking when compared to persons who primarily drank beer (OR = 0.51). While non-significant, the trend from this analysis shows persons who primarily drank beer at their primary drinking location had the highest odds for heavy drinking, except for people who primarily drank stout (OR = 1.26).

**Table 2:** Simple logistic regression of heavy drinking by primary beverage

Primary Beverage	OR	95% CI	P-Value
Beer	(ref)		
Low Alcohol Beer	0.13	0.02, 1.11	0.06
Home Brew Beer	0.41	0.04, 4.66	0.46
Stout	1.26	0.20, 7.87	0.79
Wine	0.62	0.23, 1.63	0.31
Spirits	0.45	0.18, 1.09	0.08
Cocktails	─^a^		
Liqueur	─^a^		
Shooters	─^a^		
Cider	0.51	0.29, 0.88	0.02
Alcopops	─^a^		

Simple logistic regression means univariate logistic regression

^a^All of the persons who primarily drink cocktails; liqueur; shooters; sherry, port, or vermouth; or alcopops at their primary drinking location were not heavy drinkers

Table 3 summarizes the results of the simple logistic regression predicting heavy drinking by primary drinking location. As compared to people who primarily drank in their own home, people who primarily drank at someone else’s home (OR = 2.22), nightclubs (OR = 4.58), or sports clubs (OR = 15.32) had increased odds for heavy drinking.

**Table 3:** Simple logistic regression of heavy drinking by primary drinking location

Primary Location	OR	95% CI	P-Value
Home	(ref)		
Someone Else’s Home	2.22	1.18, 4.20	0.02
Nightclub	4.58	1.25, 16.80	0.02
Sports Club	15.32	1.72, 136.81	0.02
Other Club	3.97	0.60, 26.51	0.15
Restaurant	0.28	0.04, 2.22	0.21
Motor	7.89	0.49, 127.99	0.14
Outdoors	0.71	0.13, 3.96	0.68
Shebeen	1.85	0.40, 8.50	0.41
Pub	1.55	0.72, 3.35	0.25
Special Events	13.00	0.50, 335.63	0.12
Other	─ ^a^		

^a^All of the participants who primarily drink at other locations were not heavy drinkers

Table 4 summarizes the results from the main multiple logistic regression. Heavy drinking did not differ by age, race/ethnicity, or total annual personal income after adjusting for marital status and primary container size. Persons who never married had 2.91 times the odds of heavy drinking as persons who were married, and persons who were separated have 4.45 times the odds of heavy drinking as persons who were married. Primary container size proved to have a strong association with heavy drinking. Persons who drank their primary beverage from above average-sized containers at their primary location had 7.91 times the odds of heavy drinking as compared to  persons who drank from average-sized containers.

**Table 4:** Multiple logistic regression of heavy drinking

	Heavy Drinking		
	AOR	95% CI	P-Value
Age			
18–19	0.38	0.05, 2.99	0.34
20–24	0.42	0.14, 1.22	0.11
25–34	0.70	0.30, 1.62	0.39
35–44	0.72	0.33, 1.57	0.38
45–54	0.56	0.20, 1.59	0.26
55–65	(ref)		
Race/Ethnicity			
Black African	(ref)		
Coloured	1.60	0.76, 3.39	0.20
White	0.54	0.25, 1.14	0.10
Asian/Indian	4.55	0.67, 30.82	0.11
Marital Status			
Married	(ref)		
Co-habitating	1.88	0.87, 4.06	0.10
Never married	2.91	1.57, 5.39	< 0.01
Divorced	2.66	0.65, 10.94	0.16
Separated	4.45	1.34, 14.75	0.02
Widowed	5.65	0.57, 55.68	0.13
Total Annual Personal Income			
Low	(ref)		
Medium	1.38	0.71, 2.69	0.33
High	1.41	0.39, 5.07	0.58
Container Size			
Below Average	1.00	0.34, 2.94	0.99
Average	(ref)		
Above Average	7.91	3.94, 15.86	< 0.001

### Heavy drinking by beverage type

Table 5 summarizes heavy drinking by the four main beverage types. The relationship between age and heavy drinking differed by beverage type. As compared to persons aged 55–65, persons aged 35–44 had 5.93 times the odds of heavy drinking among wine drinkers. High-income persons who primarily drank beer at their primary drinking location had higher odds of heavy drinking (AOR = 7.71). An association between heavy drinking and marital status was only present among persons who primarily drank beer at their primary drinking location. Among beer drinkers, persons who were never married had 2.44 times the odds of heavy drinking as persons who were married. There were consistently strong associations between primary container size and heavy drinking across all beverage types. Drinking from an above average-sized container predicted heavy drinking among beer drinkers (AOR = 6.94), wine drinkers (AOR = 38.26), spirits drinkers (AOR = 14,657.39), and cider drinkers (AOR = 7.52). However, persons who primarily drank beer from below average-sized containers also had increased odds of heavy drinking when compared to their counterparts who typically drank from average-sized containers (AOR = 4.02).

**Table 5:** Multiple logistic regression predicting heavy drinking by beverage type

	Beer (*n* = 384)		Wine (*n* = 74)		Spirits (*n* = 70)		Cider (*n* = 140)	
	AOR	95% CI	AOR	95% CI	AOR	95% CI	AOR	95% CI
Age								
18–19	1.10	0.01, 86.52	0.61	0.01, 44.26	─ ^d^		2.04	0.08, 53.06
20–24	0.76	0.24, 2.43	4.57	0.12, _^e^	< 0.01*	< 0.01, 0.63	0.12	0.01, 2.40
25–34	0.97	0.35, 2.67	1.33	0.13, 13.34	0.01	< 0.01, 6.54	0.14	0.01, 2.00
35–44	0.88	0.29, 2.69	5.93*	1.09, 32.28	0.01	< 0.01, 2.25	0.06	< 0.01, 1.00
45–54	0.94	0.28, 3.17	0.88	0.06, 13.16	0.03	< 0.01, 3.35	0.04*	< 0.01, 0.89
55–65	(ref)		(ref)		(ref)		(ref)	
Race/Ethnicity								
Black African	(ref)		(ref)		(ref)		(ref)	
Coloured	2.12	0.66, 6.85	0.32	0.11, 1.00	3097.26	0.89, _^e^	2.52	0.64, 9.98
White	1.17	0.18, 7.42	0.18*	0.04, 0.95	0.14	0.01, 1.35	1.32	0.29, 5.97
Asian/Indian	2.45	0.39, 15.24	─ ^b^		253.88*	1.08, _^e^	─ ^d^	
Marital Status								
Married	(ref)		(ref)				(ref)	
Co-habitating	1.24	0.53, 2.91	2.87	0.22, 38.00	7.68	0.59, 99.50	3.35	0.46, 24.25
Never married	2.44*	1.11, 5.38	2.45	0.21, 28.61	6.87	0.45, _^e^	2.25	0.65, 7.82
Divorced	0.12	0.01, 1.69	12.63	0.54, _^e^	0.88	< 0.01, _^e^	0.06	< 0.01, 1.04
Separated	9.27	0.94, 91.38	─ ^d^		─ ^d^		2.38	0.13, 45.32
Widowed	8.52	0.28, _^e^	5.53	0.31, 97.87			─ ^d^	
Total Annual Personal Income^b^								
Low	(ref)		(ref)		(ref)		(ref)	
Medium	2.00	0.83, 4.80	0.02	< 0.01, 1.57	1314.09	0.35, _^e^	3.61	0.87, 14.89
High	7.71*	1.57, 37.81	0.09*	0.02, 0.55	5.42	0.81, 36.39	0.86	0.11, 6.60
Container Size^c^								
Below Average	4.02*	1.03, 15.64	─ ^c^		18.01	0.40, 820.20	0.80	0.06, 10.05
Average	(ref)		(ref)		(ref)		(ref)	
Above Average	6.94***	3.07, 15.67	38.26***	8.44, _^e^	14,657.39*	6.15, _^e^	7.52**	2.23, 25.35

*p < 0.05, **p < 0.01, ***p < 0.001

^a^No participants drink below average sized containers of wine

^b^All participants in this cell are heavy drinkers

^c^No observations in this cell

^d^All participants in this cell are heavy drinkers

^e^Upper confidence interval > 100 probably due to small numbers of participants in a given response category for a particular beverage

## Discussion

A large proportion (53%) of drinkers in Tshwane Metropole, South Africa drank heavily (70% of men and 30% of women), even when using a conservative definition of heavy drinking. These heavy drinkers drank the vast majority (93.9%) of the absolute alcohol sold. Primary beverage container size emerged as having the most consistent association with heavy drinking, and it held across four of the most common beverage types. Drinkers who primarily drank from above average-sized containers had nearly 8 times the odds of heavy drinking compared to persons who primarily drank from average-sized containers after adjusting for demographics like age, sex/gender, race/ethnicity and income level. Surprisingly heavy drinking did not differ by gender (*p* = 0.06), but other studies conducted among drinkers in South Africa, have similar levels of risky drinking at weekends among male and female drinkers [29].

Overall, one of the most significant findings is that heavy drinking appears to be a common occurrence among drinkers in Tshwane Metropole. Given the level of harms associated with this drinking pattern, researchers and practitioners should place greater focus on monitoring and preventing heavy drinking because it may foreshadow needs for chronic health services. Our prevalence estimates are similar but higher than those of previous estimates from South Africa, such as 47.5% in 2002–2004 [30] and 48.2% among males and 22.8% among females in 2014–2015 [22]. They are likely to be more accurate given that the innovative location-specific alcohol consumption questions in the IAC cover 94% of taxable alcohol sales [31] while the standard quantity-frequency measures used in most alcohol research only cover 40–60% of these sales [32].

While in other IAC countries less than 70% of the absolute alcohol was consumed during heavy drinking occasions (e.g., Mongolia and Thailand reported 62% and St. Kitts and Nevis reported 57%), 93% of the absolute alcohol was consumed during heavy drinking occasions in Tshwane, South Africa [33]. South Africa’s political and economic history history, and the associated demographic nuances all provide clues to understanding this difference. At the end of apartheid, South Africa inherited a large number of informal alcohol outlets (“shebeens”) existing outside of the formally regulated business sector [34]. As informal outlets, shebeen owners were often undeterred by consequences established using the regulatory framework. In addition, shebeens were an integral part of the social fabric of South Africa, as there were often few recreational opportunities outside of these establishments. In examining the larger legislative framework, South Africa’s national alcohol policy was last revised in June 2013, and the current version contains few effective mechanisms to control the harmful use of alcoholic beverages [34]. As of mid 2018, South Africa does not have national restrictions on the days, hours, location, or density of alcohol outlets, and it used voluntary/self-regulation for most types of advertising and product placement. Other factors likely to play a role in South Africa’s extremely high levels of heavy drinking include high levels of poverty and social inequality, and experience of and exposure to interpersonal violence [35].

The results from this analysis also imply that there is another important contextualizing factor at play in the South African drinking environment: the alcohol industry. Our finding that 93.9% of the absolute alcohol is consumed by heavy drinkers in the Tshwane Metropole suggests that the alcohol industry’s revenues in South Africa depend on heavy drinking. The alcohol industry often argues that alcohol-related problems only affect a subset of drinkers and the majority of drinkers consume alcohol “responsibly” [36], but these data strongly contradict that conclusion.

Another key finding from this study is the strong association between primary container size and heavy drinking in Tshwane Metropole. This suggests that container size may promote risky drinking and that the liquor industry may well be driving heavy drinking and hence be a major contributor to alcohol’s high burden of death and disability in South Africa. Beer containers in South Africa often contain two or more standard drinks (i.e., 750 ml and 660 ml containers). In 2017, South African Breweries introduced a larger version (500 ml) of Carling Black Label beer, and priced it the same as their previous 440 ml version [37]. That same year, their launch of “Ama 1 litre” Black Label beer also introduced more affordable, large containers of beer, which are likely to be the major vehicle for beer sales in future. The authors are unaware of research about the association between beer container sizes and related harms. However, increased wine container sizes are associated with increased alcohol consumption [38] and symptoms of alcohol-related problems [39].

Despite the IAC’s innovative survey design, this analysis has limitations. These data are specific to Tshwane, and may not generalize to other parts of South Africa. The analyses only included data from adult participants, so the results may not generalize to youth drinkers. Future research should expand to other areas in South Africa to determine whether these trends are local, regional, or national, and should include youth to detect age-related trends early in life. Data for this study are cross-sectional, so we are unable to rule out reverse causation, which would suggest, for example, that heavy drinkers choose extra-large containers.

The definition of heavy drinking used in this study is extreme. Considering that the threshold of heavy drinking is designed to separate drinkers who cause/experience harm from others, such a definition may result in false negatives. However, this choice in operational definitions also increases the likelihood that the drinkers identified as problematic using this definition really are suffering/causing alcohol-related problems. Relatedly, the IAC questionnaires ask about “typical” drinking occasions, and it is possible that respondents overestimate the amount of alcohol consumed when they average across drinking events. However, this analysis capped reports of alcohol consumption at roughly 2500 g of absolute alcohol, which would limit the effects of any extreme overestimation. Further, the previous studies that demonstrate the IAC has high coverage of alcohol sales [31] suggests that this overestimation is likely small if it exists.

Finally, a sizable portion of respondents had missing data and were excluded from the analysis. The net effect of this missingness may have been to increase the width of some of our confidence intervals, rendering some differences insignificant. This may explain non-significant findings for differences in heavy drinking prevalence by sex/gender, and in the odds of heavy drinking in the simple linear regression for beverage type and the multiple regressions that stratified by beverage type.

## Conclusions

Heavy drinking is common among current drinkers in South Africa, and heavy drinkers consume most of the alcohol sold. Primary container size emerged as the most robust correlate of heavy drinking. South Africa is currently contemplating alcohol policy reform, and this study underscores the importance of these draft policies. The draft liquor amendment bill of 2016 proposes several evidence-based policies that could help reduce these heavy drinking occasions. Rigorous monitoring of the heavy drinking environment may also serve to establish baseline data to evaluate the effects of any future policy changes.

## Abbreviations

AA: Absolute alcohol
AOR: Adjusted odds ratio
AUD: Alcohol use disorder
CI: Confidence interval
EA: Enumeration area
g: Gram
IAC: International Alcohol Control
ml: Milliliters
OR: Odds ratio
